# Research progress on transcranial magnetic stimulation for post-stroke dysphagia

**DOI:** 10.3389/fnbeh.2022.995614

**Published:** 2022-08-18

**Authors:** Yi Li, Kerong Chen, Jiapu Wang, Hanmei Lu, Xiaoyu Li, Lei Yang, Wenlu Zhang, Shujuan Ning, Juan Wang, Yi Sun, Yu Song, Mei Zhang, Jianhong Hou, Hongling Shi

**Affiliations:** ^1^Department of Rehabilitation Medicine, Third People’s Hospital of Yunnan Province, Kunming, China; ^2^Department of Orthopedics, Third People’s Hospital of Yunnan Province, Kunming, China

**Keywords:** post-stroke dysphagia, neuroplasticity, transcranial magnetic stimulation, effectiveness, therapeutic mechanism

## Abstract

Dysphagia is one of the most common manifestations of stroke, which can affect as many as 50–81% of acute stroke patients. Despite the development of diverse treatment approaches, the precise mechanisms underlying therapeutic efficacy remain controversial. Earlier studies have revealed that the onset of dysphagia is associated with neurological damage. Neuroplasticity-based transcranial magnetic stimulation (TMS), a recently introduced technique, is widely used in the treatment of post-stroke dysphagia (PSD) by increasing changes in neurological pathways through synaptogenesis, reorganization, network strengthening, and inhibition. The main objective of this review is to discuss the effectiveness, mechanisms, potential limitations, and prospects of TMS for clinical application in PSD rehabilitation, with a view to provide a reference for future research and clinical practice.

## Introduction

Dysphagia, defined as “difficulty swallowing,” is one of the most important clinical manifestations of stroke and a common consequence of neurological damage caused by a range of diseases (Fung et al., 2004). Studies have confirmed that 50–81% of acute stroke patients may experience swallowing problems (Hamdy, 2010). In most cases, the post-stroke dysphagia (PSD) will improve spontaneously. However, approximately 11–50% of patients may have long-term disability (Kumar et al., 2010; Cohen et al., 2016).

Although dysphagia gradually resolves spontaneously in the early stages of disease in most cases, severe and persistent forms of dysphagia remain prevalent in about 13% of stroke patients (Mann et al., 1999). The presence of dysphagia is linked to increased physical and psychological stress in patients, families, and caregivers, along with reduced quality of life (Eslick and Talley, 2008). In addition, dysphagia may cause various life-threatening complications, such as aspiration pneumonia, asphyxia, dehydration, and malnutrition (Smithard et al., 1996). In particular, aspiration pneumonia can trigger various complications, the most acute being infection and sepsis (Kalita et al., 2015). These complications increase the risk of prolonged hospital stays, high medical expenses and significant mortality, causing a major negative impact at both the individual and society level. Therefore, rehabilitation therapy of PSD remains a significant clinical issue that needs to be urgently addressed.

Studies have demonstrated that central causes of dysphagia in stroke patients include cortical or brain-stem damages, and peripheral causes include damages to the nerves or muscles involved in swallowing. The brain-stem lesions are more commonly associated with dysphagia (Balcerak et al., 2022). Notably, dysphagia is usually caused by infratentorial lesions, while sensory afferent disturbances usually cause dysphagia in supratentorial stroke. However, the exact mechanism of PSD is not well understood.

The treatment options of PSD include behavioral therapy, oral care, pharmacology, neurostimulation, and dietary interventions. Various physical therapies and preventive measures can avoid dysphagia-related complications. However, there is a lack of medical or electrophysiological interventions to facilitate recovery from dysphagia after acute or subacute stroke.

Existing treatments for PSD include postural training (Hägg and Larsson, 2004), dietary modification (Hägg and Anniko, 2008; McCullough et al., 2012), swallowing movements (Hägg and Anniko, 2008), compensation techniques (Lin et al., 2003), drug therapy, oral motor stimulation (Kang et al., 2012), music therapy (Kim, 2010), facial sensory stimulation, pharyngeal electrical stimulation, neuromuscular electrical stimulation, non-invasive brain stimulation, botulinum toxin injection, and acupuncture therapy (Terré et al., 2013; Yang et al., 2015). Nevertheless, these treatment strategies cannot change the physiology of impaired swallowing biomechanics as well as cannot promote the recovery of impaired swallowing neural networks in stroke patients (Speyer et al., 2010).

According to a previous study, the pathogenic cascade of dysphagia is as follows: after peripheral or central (corticobulbar tract) impairment of the cranial nerves innervating the swallowing muscles, tongue movement is limited, with soft palate paralysis. Consequently, intraoral and pharyngeal pressure cannot be fully increased, movement of food from the oral cavity to the pharynx and esophagus is weak, and transit time is significantly prolonged. The retention increases hyperreflexia or spasm of sphincter and cricopharyngeal muscle in the esophageal inlet of patients with supraglomerular damage (pseudobulbar palsy) and movement of the swallowing muscles is uncoordinated, resulting in accidental ingestion of food into the trachea (Ertekin et al., 2000). In recent years, accumulating evidence has shown that transcranial magnetic stimulation (TMS) can induce changes in the excitability of the cerebral cortex, promote plastic alterations in nerves, control the release of neurotransmitters (Lanza et al., 2015), and manage dysphagia through regulating neuroplasticity. The main objective of this review is to synthesize clinical studies and investigate the effectiveness, mechanisms of action, advantages, and disadvantages of TMS in clinical practice.

## Transcranial magnetic stimulation

Transcranial magnetic stimulation is a non-invasive stimulation technique based on the principles of neuroplasticity that induces changes in neurological pathways by altering neurons in target cortical areas through synaptogenesis, reorganization, network strengthening, and inhibition, causing local depolarization of the magnetic field below the skull and activation or inhibition of activity in cortical areas (Hallett, 2000; Koerselman et al., 2004). It was also reported that the feasibility of using external magnetism to stimulate the nerves and brain (Barker et al., 1985). The group described TMS as a non-invasive technique to stimulate the human motor cortex. At present, TMS is widely used as a routine diagnostic tool in neurophysiological studies owing to its safe and technical characteristics (Rossi et al., 2009). This approach is based on speech, language, and swallowing disorders of the nervous system (Naeser et al., 2005; Khedr et al., 2009; Verin and Leroi, 2009; Barwood et al., 2011a,b,c). TMS exerts therapeutic effects by directly modulating specific pathways in the brain, which may ultimately affect longer-term communication and swallowing outcomes. Recent advances in TMS technology facilitate its application in clinical neurorehabilitation programs for patients with brain injury. Earlier reports have also demonstrated positive therapeutic effects on swallowing function after TMS, highlighting its potential as a treatment modality for dysphagia (Ridding and Rothwell, 2007). Multiple systematic reviews and meta-analyses have confirmed the beneficial effects of TMS on PSD (Yang et al., 2015; Pisegna et al., 2016; Liao et al., 2017; Chiang et al., 2019; Marchina et al., 2021) and swallow-related outcomes in patients. Moreover, the most intense effects of peripheral and cortical neurostimulation, including those of TMS, occur during the first 2 weeks after stroke (Yang et al., 2015). The efficacy of TMS for PSD from clinical trials and meta-analyses were illustrated in Table 1.

**TABLE 1:** Summary of studies on the efficacy of TMS for PSD from clinical trials and meta-analyses.

Stimulation mode and intensity	Stimulation target	Sample	Treatment cycle	Test method	Main results	References
rTMS (3 Hz)	Target cortical representation in ipsilateral pharyngeal region	21	5 days	WST	rTMS > basic rehabilitation training; improvement rates of the control and rTMS groups were 31.0 and 65.6%, respectively; WST score; the standard, improvement of dysphagia in the rTMS group was significantly higher than that in the control group (*p* < 0.05)	Yang et al., 2015; Jiao et al., 2022
rTMS (10 Hz)	Bilateral irritation	35	3 weeks	CDS, DOSS, PAS, VDS	CDS, DOSS, PAS, and VDS scores in both groups; scores in the bilateral group > scores in the unilateral group (*p* < 0.05)	Park et al., 2017
rTMS (10 Hz)	Ipsilateral motor cortex	35	3 weeks	CDS, DOSS, PAS, VDS	CDS, DOSS, PAS, and VDS scores in both groups; scores in the bilateral group > scores in the unilateral group (*p* < 0.05)	Park et al., 2017
TMS (5 Hz)	Lingual cortical motor area	15	10 days	VFSS, SAPP	No significant difference in VFSS or SAPP were observed between the two groups	Cheng et al., 2017
TMS (3 Hz)	Ipsilateral	15	5 days	WST, DD, cortical excitability	Both WST and DD were improved as well as cortical excitability in the affected hemisphere	Du et al., 2016
TMS (1 Hz)	Contralateral	13	5 days	WST, DD, cortical excitability	Both WST and DD were improved as well as cortical excitability in the unaffected hemisphere and cortical excitability in the affected hemisphere	Du et al., 2016
rTMS (10 Hz)	Ipsilateral	16	10 days	SSA, DD, cortical excitability	Cortical excitability in the affected or unaffected hemisphere were improved; significant improvement in SSA score; no change in DD score	Zhang et al., 2019
rTMS (1 Hz)	Contralateral	16	10 days	SSA, DD, cortical excitability	Cortical excitability in the affected or unaffected hemisphere were improved; significant improvement in SSA score; no change in DD score	Zhang et al., 2019
rTMS (1 Hz)	Epilepsy	16	10 days	SSA, DD, cortical excitability	Cortical excitability in the affected or unaffected hemisphere were improved; SSA score in the bilateral group > SSA score in the unilateral group; no change in DD score	Zhang et al., 2019
rTMS (1 Hz)	Contralateral	6	15 days	MASA and Functional Oral Intake Scale	MASA and functional oral intake scale scores were improved	Tarameshlu et al., 2019
TMS (3 Hz)	Ipsilateral esophageal cortical area	14	5 days	DD	Improvement in DD score	Khedr et al., 2009
rTMS (10 Hz)	Contralateral motor cortex of bilateral mylohyoid muscles	11	2 weeks	CDS, DOSS, PAS, VDS	DOSS, PAS and VDS scores in the bilateral group > scores in the unilateral group	Park et al., 2017
rTMS (10 Hz)	Ipsilateral motor cortex of mylohyoid muscle	12	2 weeks	CDS, DOSS, PAS, VDS	DOSS, PAS and VDS scores in the bilateral group > scores in the unilateral group	Park et al., 2017
rTMS (1 Hz)	Ipsilateral	4	5 days	MASA	MASA scores were improved	Ghelichi et al., 2016
rTMS (5 Hz)	Ipsilateral pharyngeal motor hotspot	8	2 weeks	PAS, VDS	VDS score: significant improvement in pharyngeal motor function. Activation of bilateral primary motor cortices, anterior motor cortex, and right prefrontal cortex	Park et al., 2019
rTMS (5 Hz)	Lingual motor cortex	2	2 weeks	MASA and swallowing-related quality of life	MASA and swallowing-related quality of life were improved	Cheng et al., 2015
rTMS (10 Hz)	Cerebellum	1	/	PMEP, cPAS	Improvement in PMEP amplitude (55% above baseline) and swallowing safety (17% below baseline)	Vasant et al., 2019
rTMS (1 Hz)	Contralateral	14	4 weeks	MASA and quality of life assessments	Improvement in quality of life; no significant change in MASA	Ünlüer et al., 2019

TMS, transcranial magnetic stimulation; PSD, post-stroke dysphagia; WST, water-swallowing test; CDS, Clinical Dysphagia Scale; DOSS, Dysphagia Outcome and Severity Scale; PAS, Permeation Aspiration Scale; MASA, Mann Assessment of Swallowing Ability; VDS, Videofluoroscopic Dysphagia Scale; PMEP, representative pharyngeal motor evoked potential amplitude; VFSS, videofluoroscopic swallowing study; SAPP, swallowing activity and participation profile; cPAS, cumulative penetration-aspiration score; DD, degree of dysphagia; SSA, standardized swallowing assessment.

### Mechanism of action of transcranial magnetic stimulation

Transcranial magnetic stimulation, a tool for high-pressure brain stimulation, presents an alternative method for treatment of dysphagia *via* modulation of neuroplasticity. The procedure is based on the principle of inductance and non-invasively transmits electrical energy to the brain through the scalp and skull (Wassermann, 1998). A large current pulse generator is employed to release high currents thousands of amperes greater than that flowing through the coil, up to several kilowatts in power. These short magnetic pulses cause a sustained increase or decrease in cortical excitability. A brief but intense current is passed through a TMS coil placed on the scalp, creating a magnetic field that penetrates the skull to a depth of about 1.5–2 cm and induces a sufficiently strong electric field to depolarize surface axons and activate cortical neural networks (Lefaucheur et al., 2014). In addition, an electromyographic response to the target musculature is produced, known as motor-evoked potential (MEP) (Fitzgerald et al., 2006). Subsequently, descending motor shooting along the corticospinal tracts from the cortex to peripheral muscles is elicited to adjust the excitability of the cerebral cortex. TMS can be divided into high frequency (≥1 Hz) TMS and low frequency (≤1 Hz) stimulation processes (Wassermann, 1998). High frequency tends to enhance the excitability of the cerebral cortex while low frequency exerts the opposite effect (Hamdy et al., 1998; Fitzgerald et al., 2006). In stroke patients recovering from dysphagia, functional recovery was found to be associated with increased cortical representation of the intact hemisphere, highlighting the importance of reorganization of intact neural networks in PSD recovery (Pascual-Leone et al., 1998). Repetitively applied TMS, also known repetitive TMS (rTMS), can induce changes in synaptic plasticity similar to long-term potentiation (LTP) or long-term depression (LTD), that is, increased or decreased synaptic strength (Stefan et al., 2002; Hoogendam et al., 2010). The precise mechanism remains unknown but is thought to be mediated by the activity of *N*-methyl-D-aspartate (NMDA) receptors, as revealed by studies using NMDA antagonists (Fitzgerald et al., 2006; Huang et al., 2007). Other known rTMS modalities include intermittent (excitatory) theta burst stimulation (iTBS) and continuous (inhibitory) TBS (cTBS) (Ridding and Rothwell, 2007). However, recent reports suggest that the ability to respond to these protocols varies on an individual basis (Ridding and Rothwell, 2007).

Studies have confirmed that the damages to subcortical white matter (the internal capsule and within the brainstem) caused dysphagia, possibly due to disruption in the sensorimotor pathways of the corticobulbar tract. TMS may exert effects on PSD by regulating sensorimotor pathways in the brain. However, the details of how TMS change the communication and connection of cortical neural networks to achieve the therapeutic effect remain largely unexplored. The advantages and disadvantages of TMS were shown in Table 2.

**TABLE 2:** Advantages and disadvantages of transcranial magnetic stimulation (TMS).

Advantages	Disadvantages
➀ Good safety and non-invasiveness	➀ No significant beneficial effects on genetic factors, death, dependence, disability, prognosis and length of hospital stay
➁ Long-term impact on communication and swallowing disorder prognosis	➁ The effect of nerve stimulation therapy was not analyzed separately
➂ Potentially improves performance after administration	➂ The number of studies is limited, with small sample sizes, uneven case quality, and heterogeneity among studies
➃ TMS can enhance muscle control of swallowing after stroke	
➄ Shorter course of treatment	
➅ TMS induces alterations in the functional status of local cerebral cortex, enhances synaptic function, and regulates neuronal function in the brain	
➆ Accurate and optimal balance in the excitatory and inhibitory control functions of the cerebral cortex	

### Advantages of transcranial magnetic stimulation

Transcranial magnetic stimulation is widely regarded as a safe and non-invasive form of nerve stimulation that can be used to directly manipulate cerebral cortex activity. In recent years, this innovative neuromodulation technology has been widely applied in neuroscience and countless cognitive fields (Barwood et al., 2011b) and shown to exert therapeutic effects by directly regulating specific pathways in the brain, which could ultimately affect longer-term communication and swallowing disorder prognosis (Naeser et al., 2005; Cotelli et al., 2008; Khedr et al., 2009; Verin and Leroi, 2009; Barwood et al., 2011a,c; Geeganage et al., 2012; Murdoch et al., 2012). The potential nerve priming effect induced by TMS is reported to effectively improve performance. Recent progress in TMS technology facilitates its application in clinical neurorehabilitation programs for patients with brain injury and the existing evidence shows that high-frequency TMS can enhance the muscle control of swallowing after stroke. For instance, in a study by Verin and Leroi (Geeganage et al., 2012) using TMS to stimulate the musculohyoid cortical area, the swallowing function of the patient improved at 3 days after stimulation. In a review by Cochrane (Zhai et al., 2020) on management of PSD, cortical rTMS reduced the need for physical or cognitive engagement in complex cases and had the potential to shorten the course of treatment. Previous studies have demonstrated that this non-reduced magnetic signal can reach the target area of brain tissue through the skull, thereby changing the functional status of the local cerebral cortex, enhancing synaptic function, and regulating neuronal function in the brain (Bath et al., 2018). Moreover, TMS has different intensities, frequencies and stimulation areas and can modulate the relationships and interactions among neural networks, thus affecting the functions of different regions. TMS promotes accurate and optimal balance of excitatory and inhibitory control functions in the cerebral cortex.

### Current limitations

Despite the considerable benefits of TMS, lots of limitations restrict its use in clinical practice in terms of effectiveness, safety, and clinical study design. First, no significant beneficial effects of TMS on genetic factors, death, dependence, disability, prognosis, or length of hospital stay have been reported (Hoshi and Tamura, 1993; Wiethoff et al., 2014; Horvath et al., 2016). Second, patients in a few of earlier trials received traditional rehabilitation training, which made it impossible to separately analyze the effects of nerve stimulation therapy. In treatment of PSD with TMS, the optimal choice of stimulation site (unaffected hemisphere, affected hemisphere, or bilateral hemispheres) has not yet been determined. Based on different viewpoints on the recovery mechanism of PSD, the choice of excitatory stimulation (high frequency) or inhibitory stimulation (low frequency) at the corresponding site (involved, affected, or bilateral side) is controversial. In additions, the number of reported studies is limited, with small sample sizes, uneven case quality and significant heterogeneity among studies. Therefore, the available data are insufficient draw accurate conclusions on the recommended optimal treatment regimen.

## Future prospects

This review provides a summary of the efficacy and underlying mechanisms of TMS activity in patients with PSD. A large majority of studies to date has used water-swallowing test (WST), clinical dysphagia scale (CDS), Dysphagia Outcome and Severity Scale (DOSS), Permeation Aspiration Scale (PAS), Mann Assessment of Swallowing Ability (MASA), Videofluoroscopic Dysphagia Scale (VDS), representative pharyngeal motor evoked potential (PMEP) amplitude, cumulative penetration-aspiration score (cPAS), and degree of dysphagia (DD) to evaluate the significance of the results. However, given the evidence for the validity of the results, it may be possible to incorporate more credible tests to draw strong conclusions in future studies. In 1993, Hoshi and Tamura demonstrated the validity of measuring different cortical regions with functional near-infrared spectroscopy (fNIRS). For the first time, the potential of fNIRS imaging brain activation sequences were reported (Ehlis et al., 2009). fNIRS is a neuroimaging technique used to map the function of the human cerebral cortex that utilizes the principle of near-infrared (NIR) spectroscopy (NIRS). Changes in optical properties of the human cerebral cortex are detected simultaneously from multiple measurement sites and the results displayed in the form of maps or images in specific areas. Over the years, fNIRS has emerged as a key neuroimaging technique that has contributed significantly to advances in understanding human brain function. In recent years, the validity of fNIRS measurements has been repeatedly demonstrated by simultaneous functional magnetic resonance imaging (fMRI) measurements, with widely recognized applications in newborn/child and adult language processing in cognitive neuroscience. Although TMS demonstrate great potential to accelerate the improvement of swallowing function in patients with PSD, there is currently a lack of real-time assessment tool for brain function to optimize TMS parameters. As an assessment tool of brain activity, fNIRS can be used to measure the changes in hemoglobin (Hb) concentrations within the brain, which can not only evaluate the effect of TMS treatment, but also can guide the optimization of TMS treatment regimen during the PSD rehabilitation. In the future, we should combine the TMS and fNIRS to serve as a reference for upcoming clinical and laboratory research.

## Author contributions

All authors listed have made a substantial, direct, and intellectual contribution to the work, and approved it for publication.
